# High-resolution bathymetry by deep-learning-based image superresolution

**DOI:** 10.1371/journal.pone.0235487

**Published:** 2020-07-01

**Authors:** Motoharu Sonogashira, Michihiro Shonai, Masaaki Iiyama

**Affiliations:** 1 Academic Center for Computing and Media Studies, Kyoto University, Kyoto, Kyoto, Japan; 2 Ecomott Inc., Sapporo, Hokkaido, Japan; University of Florida, UNITED STATES

## Abstract

Seafloor mapping to create bathymetric charts of the oceans is important for various applications. However, making high-resolution bathymetric charts requires measuring underwater depths at many points in sea areas, and thus, is time-consuming and costly. In this work, treating gridded bathymetric data as digital images, we employ the image-processing technique known as superresolution to enhance the resolution of bathymetric charts by estimating high-resolution images from low-resolution ones. Specifically, we use the recently-developed deep-learning methodology to automatically learn the geometric features of ocean floors and recover their details. Through an experiment using bathymetric data around Japan, we confirmed that the proposed method outperforms naive interpolation both qualitatively and quantitatively, observing an eight-dB average improvement in peak signal-to-noise ratio. Deep-learning-based bathymetric image superresolution can significantly reduce the number of sea areas or points that must be measured, thereby accelerating the detailed mapping of the seafloor and the creation of high-resolution bathymetric charts around the globe.

## Introduction

**Fig 1:**
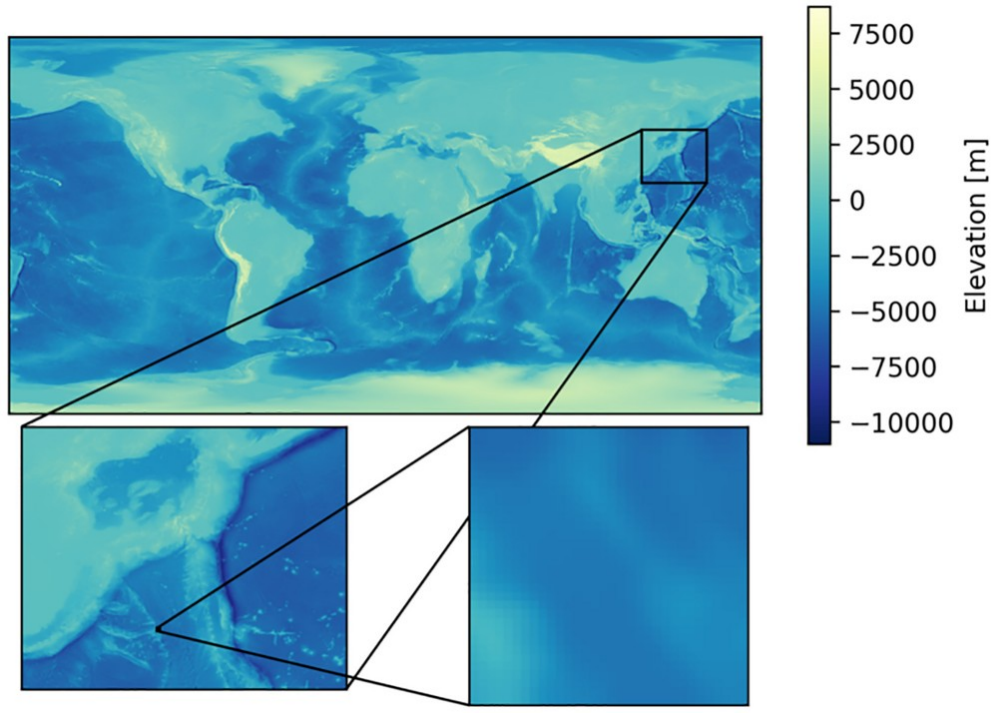
The GEBCO 30-arc-second gridded bathymetric dataset. Although it covers the oceans all over the globe, its resolution is low and each sea area is sampled coarsely, as seen in the bottom right closeup.

Bathymetric charts of the oceans describe the underwater depths of ocean floors. Today, they have a wide range of applications, e.g., ship navigation, submarine infrastructure (e.g., pipeline and cable) construction, fishery resource protection, seabed resource (e.g., mineral, oil, and gas) exploration, and seismic hazard (e.g., earthquake and tsunami) assessment. Thus, the creation of bathymetric charts is an important topic in oceanography, and seafloor mapping for this purpose has a long history. For example, the General Bathymetric Chart of the Oceans (GEBCO) project [1] has been attempting to create global terrain models of the oceans and lands for more than 100 years. However, according to the Seabed 2030 project [2], whose aim is to integrate all available bathymetric data into an improved global seafloor map, more than 80 percent of the seafloor is yet to be mapped. Among the various factors that affect the quality of a bathymetric chart, the resolution determines how much information on seafloor details the chart can contain, which is critical to its usefulness. For example, the GEBCO gridded bathymetric data are available all over the globe; however, their resolution is limited and many sea areas are sampled quite coarsely, as seen in Fig 1. Thus, it would be highly desirable to develop a new technology enabling us to create high-resolution bathymetric charts efficiently.

Traditionally, bathymetric surveying for seafloor mapping involves chartering ships equipped with specialized (often expensive) hardware such as single-beam and multi-beam sonar systems, going to targeted sea areas, and measuring the underwater depths of ocean floors as widely and densely as possible. However, despite advancements in measurement technology, this procedure is still time-consuming and costly because a ship can measure only a single point at a time (or at most a number of neighboring points in the case of multi-beam sonar). Therefore, it is obviously impracticable to measure every sea area in detail, and consequently, high-resolution bathymetric charts have been available only in limited parts of the world’s oceans.

**Fig 2:**
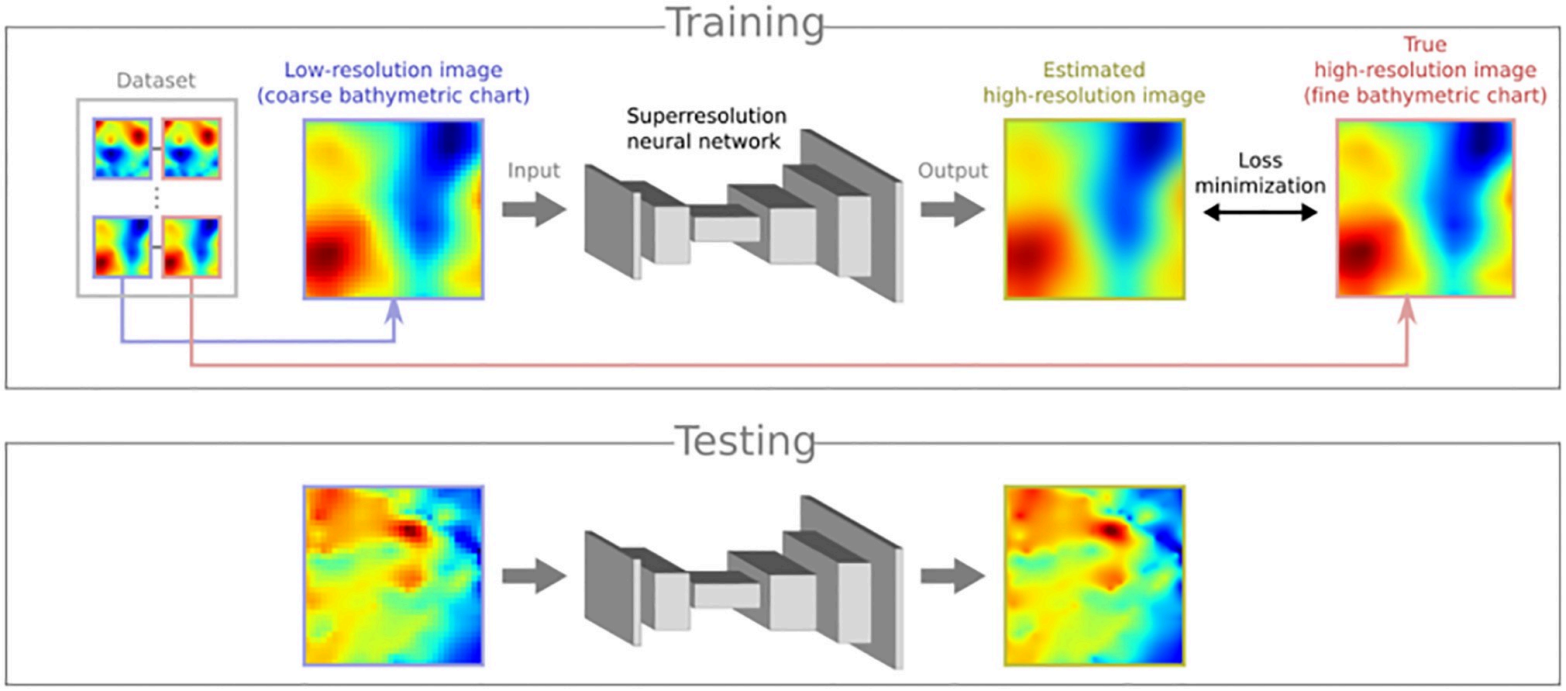
Overview of the proposed deep-learning-based image superresolution. We use a deep neural network for superresolution that takes a low-resolution image as input and yields a high-resolution image as output, which in our case represent coarse and fine bathymetric charts, respectively. First, in the training phase, we let the network learn how to estimate the high-resolution image from the low-resolution one, using a dataset consisting of many pairs of low- and high-resolution images. This is done by minimizing a loss function, which is defined as the difference between the estimated high-resolution image and the true high-resolution image corresponding to the low-resolution one. Then, in the testing phase, we can let the network predict a desired, unknown high-resolution image from each newly-given low resolution image.

In this work, we propose a novel approach to the efficient creation of high-resolution bathymetric charts. An overview of the proposed method is shown in Fig 2. Our basic observation is that a gridded bathymetric chart of a sea area can be treated as a digital image consisting of pixels whose values represent depths. Motivated by this observation, we employ a technique of digital image processing called *superresolution*, whose aim is to enhance the resolution of images by recovering missing details. In this way, we can effectively obtain fine, high-resolution bathymetric charts from coarse, low-resolution data, which are easier to obtain than high-resolution data. Thus, we can significantly reduce the number of sea areas or points that must be measured, thereby accelerating the detailed mapping of the seafloor and the creation of high-resolution bathymetric charts around the globe. More specifically, we employ a modern superresolution methodology based on deep learning to automatically extract geometric features of bathymetric images, i.e., complex structures like peaks and valleys, and accurately recover them via reconstructing high-resolution images from low-resolution ones. As our experimental results demonstrate, the proposed method is more accurate and thus, more effective than naive interpolation for resolution enhancement both in qualitative and quantitative terms. To sum up, we apply deep-learning-based image superresolution to bathymetry and reveal its effectiveness, which is the main contribution of this work.

The rest of this paper is organized as follows: first, we review related work on image superresolution (the Related work section). Then, we describe the proposed method of deep-learning-based superresolution for bathymetric images (Method). Furthermore, we demonstrate the effectiveness of the proposed method using experimental results ((Results and discussion). Finally, we conclude this paper with some comments on future work (Conclusion).

## Related work

Superresolution is an image-processing technique that restores high-resolution images from low-resolution ones. From the viewpoint of signal processing, this is equivalent to recovering the missing high frequencies from a signal that contains only low frequencies. Such a technique is needed because a digital image composed of discrete pixels arranged on a regular grid can only hold frequency information below the Nyquist frequency, i.e., half of its sampling rate. This restoration is called an inverse problem, as we attempt to invert the degradation process that lowered the resolution of an original high-resolution image. To perform this inversion, we need to provide sufficient information about the latent high-resolution image to be restored, be it additional observation or knowledge, since the high-frequency information had already been lost during the degradation and cannot be inferred from a single observed low-resolution image. Simplistic approaches like geometric interpolation can only increase the sampling rate but cannot recover the lost high-frequency details, as they fully rely on the remaining low-frequency components.

One traditional approach to the superresolution problem is multi-image superresolution [3], which uses multiple low-resolution images and integrates them to reconstruct a single high-resolution image, recovering high-frequency information by combining all the observations. These images must have slightly different contents, and are typically observed by placing multiple cameras at different locations or by moving a camera during capture. Although this approach can become quite accurate as the number of observations increases, observing multiple images can be costly and troublesome in practice and also requires calibration between cameras or registration between images, whose accuracy limits the performance of superresolution reconstruction. An alternative approach is single-image superresolution [4], which uses only a single low-resolution image and exploits prior knowledge regarding a targeted image domain (typically, natural images such as photographs) to recover high-frequency details. This approach has a lower observation demand, and thus, is easier to use in reality, provided that appropriate prior information is available. In this work, we employ the single-image approach to minimize the required amount of bathymetric data, which is more difficult to obtain in high resolution than natural images.

Traditionally, the prior knowledge required for superresolution has been manually crafted, i.e., represented as mathematical models by experts, exploiting typical characteristics of natural images, such as local smoothness between neighboring pixels (except for edges) and nonlocal similarity between patches. This approach is inherently limited by the quality of the hand-crafted priors, due to the difficulty of modeling all possible images in the real world. To overcome this limitation, learning-based superresolution has been developed, which can automatically extract effective prior knowledge from existing data by analyzing their patterns using a statistical framework. The ability of a learning-based method depends on the flexibility of the mathematical model used to estimate high-resolution images from low-resolution ones. The parameters of the model should be numerically adjusted to a problem-specific dataset consisting of samples, i.e., the pairs of low- and high-resolution images as input and expected output, respectively. This process lets the model “learn” the dataset to be able to estimate a high-resolution image from its low-resolution version, in preparation for making predictions on new samples. While in principle the performance of a flexible model increases as more data become available, very large datasets have proven been both time-consuming and difficult to learn for traditional techniques. Recently, in order to learn a large amount of data effectively and efficiently, several studies introduced the modern machine-learning framework of deep learning to superresolution, which uses deep neural networks consisting of many layers as learning models. The use of deep neural networks in superresolution was pioneered by SRCNN [5]. It is a simple convolutional neural network (CNN), where multiple convolution layers that perform image filtering operations are stacked to realize a complex image transformation. The actual types of filters (e.g., blurring and edge extraction) used by these layers depend on their parameters, which should be adjusted via learning. The resulting transformation implicitly extracts image features such as geometric structures through the iterative filtering process and matches them with learned image patterns, thereby recovering high-frequency details effectively. The learning ability of this network was further improved with more layers as DCSCN [6]. Recently, generative adversarial networks (GANs), which combine a generator (superresolution estimator) and a discriminator (image-quality evaluator) to produce more realistic images, have been introduced to deep-learning-based superresolution. While the first GAN-based network was SRGAN [7], it was later modified into ESRGAN [8], which reportedly scored the best performance in a region of the PIRM2018 Superresolution Challenge [9], a performance competition among superresolution methods. Unlike shallow networks proposed earlier [5], ESRGAN is a very deep network consisting of many layers and thus effective in modeling complex transformations, while circumventing the difficulty in learning due to the increased number of parameters by taking a residual learning approach, which reduces information to be learned at each layer and thereby facilitates smooth learning. In this work, we employ only the generator part of ESRGAN in order to benefit from its state-of-the-art performance, i.e., high accuracy given a limited amount of learning data owing to the deep residual architecture. Meanwhile, we omit the GAN scheme, and thus, do not use the discriminator, since GAN does not always produce high-resolution images faithful to low-resolution inputs, sometimes producing artifacts (e.g., fake structures) and increasing errors with respect to ground truth [10]. Since bathymetric images are more smooth than natural images, we can achieve high-quality superresolution without GAN, as we will see in the Results and discussion section. It is important to note that we cannot utilize the existing already-learned superresolution estimators without alteration because bathymetric images have different features from natural images. Hence, we let the adopted network learn our dataset of bathymetric images from scratch. Moreover, since high-resolution data are more difficult to obtain in bathymetry than in the case of natural images, we introduce data-preprocessing techniques to deal with the limited amount of available bathymetric data (see the Data preprocessing section).

To the best of our knowledge, this work is the first application of deep learning, i.e., modern machine learning using deep neural networks, to bathymetric image superresolution. However, a previous study did investigate the use of image superresolution for bathymetric data using traditional (i.e., non-deep) learning [11, 12]. The authors of this previous study compared several traditional learning techniques and found that the one called random forest was the most effective for bathymetric image superresolution [11]. They then examined its performance in greater detail [12]. However, the validity of their argument is yet to be confirmed since there seem to be no peer-reviewed papers so far (only a poster [11] and a technical report [12] are available). Unlike the previous work, which employed traditional learning techniques, we make full use of the modern learning methodology, i.e., deep learning, in order to realize more effective bathymetric image superresolution. Note that we are not able to directly compare the proposed method with their method since they have not released their code or dataset; nevertheless, we followed their experimental scheme, and consequently observed better performance (see the Results and discussion section).

## Method

Single-image superresolution is formalized as the problem of estimating a latent high-resolution image from an observed low-resolution image. Since it is difficult to manually craft a mathematical estimation model for all possible bathymetric images in the real world, we resort to the learning-based approach to superresolution, which can be used to extract the required prior information for the high-resolution image from existing data. To this end, we need to prepare a dataset made of a number of samples, each of which is a pair of low- and high-resolution bathymetric images. Since the quality of the dataset matters to the effectiveness of learning, we apply data-preprocessing techniques to the dataset (the Data preprocessing section). As a superresolution estimator, we use a deep neural network as a black-box function that transforms the low-resolution image (input) into the high-resolution image (output). The architecture of this deep network consists of many layers of several types, which are chained from the input to the output and perform various operations on the data passing through the network (Network architecture); thus, the network is flexible enough to represent a complex image transformation and able to effectively learn a large amount of data. The actual transformation performed by this network is automatically determined from the dataset during training (Training). More specifically, we adjust parameters of the network in order to ensure that the high-resolution output image estimated from each low-resolution input image in the dataset is as close to the corresponding true high-resolution one as possible. This is done via numerical optimization that minimizes a loss function, which represents the total error between the true and estimated high-resolution images. The construction and training of the network can be efficiently implemented using currently-available programming libraries (Implementation).

### Data preprocessing

In principle, the more available samples there are, the more effective a deep learning method will be. In our case, however, it is difficult to collect as many bathymetric images as natural images used in general-image superresolution. Hence, we apply two preprocessing techniques to the dataset in order to deal with the scarcity of bathymetric images available for learning. Here, we make a few assumptions about the bathymetric images, which might not hold perfectly true in reality; nevertheless, the effectiveness of these techniques will be empirically validated in the Implementation section.

**Fig 3:**
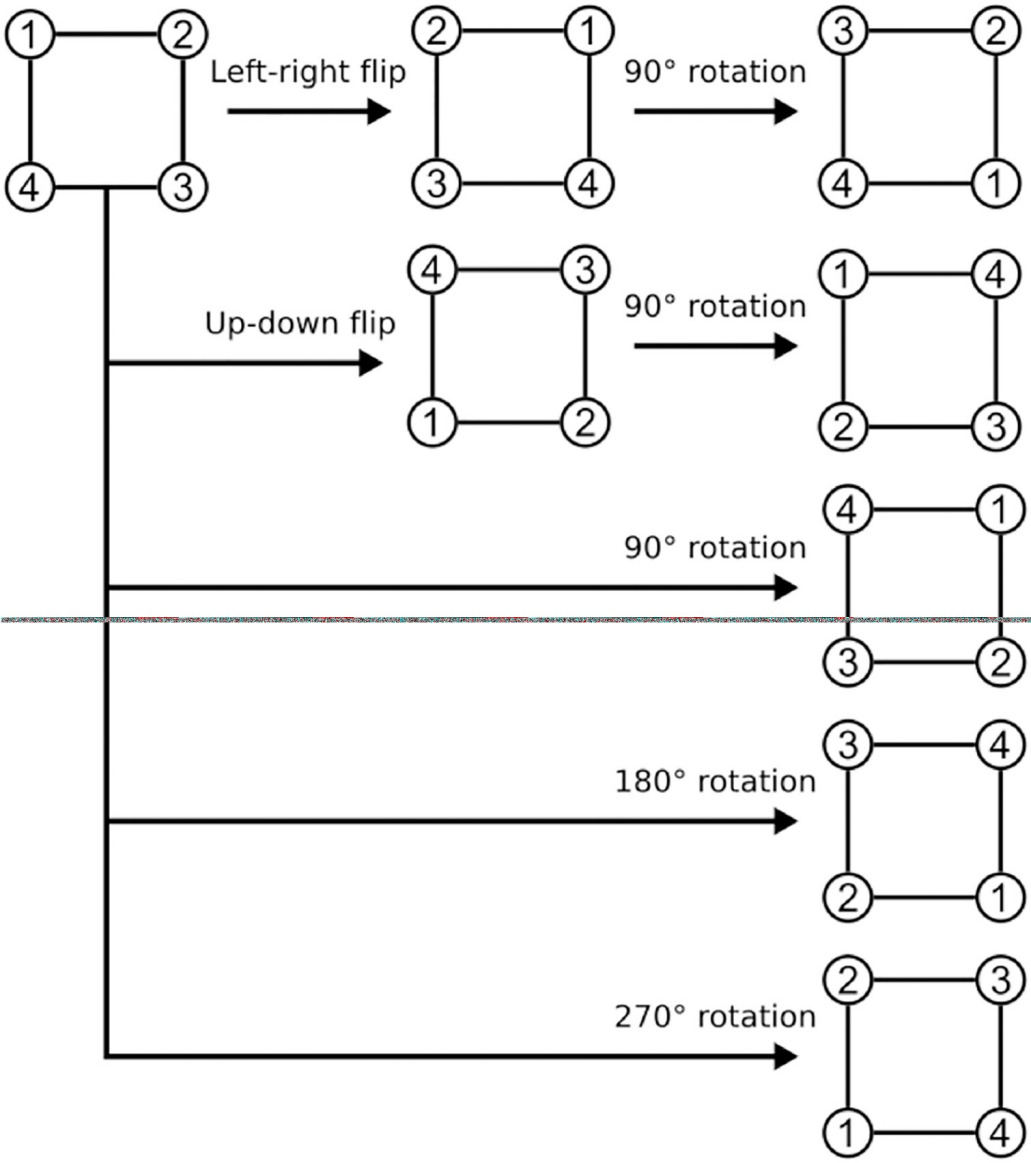
Data augmentation by flipping and rotation. The circled numbers identify image corners. There are eight possible transformations, which can be obtained by combining the flips in the left-right and up-down directions, and the rotations by 90, 180, and 270 degrees.

The first technique is data augmentation, which is a common practice in deep learning. The basic idea of augmentation is to virtually increase the number of samples by applying some transformations to the existing samples. Observing that bathymetric images are basically invariant under flipping and rotation (that is, an ocean floor still looks like an ocean floor even if viewed from different directions), we can flip and rotate the paired images in each sample. Here, we only use rotations by multiples of 90 degrees to avoid image padding, which is required when using the outer parts of images. Since there are eight possible ways to combine flipping (up-down and left-right) and rotation (90, 180, and 270 degrees), as depicted in Fig 3, we can increase the number of samples by a factor of eight.

The second technique is normalization, which compensates for the differences in value ranges between samples. This effectively reduces the variance of the dataset, and hence, the amount of information that the network needs to learn, thereby making the learning easier and the required number of samples smaller. This technique is particularly important for bathymetric images, since their depth value ranges vary a lot, depending on whether sea areas are deep or shallow, and smooth or rough. By focusing on the structures (e.g., peaks and valleys) independent of depth, we can ignore the absolute depth of each sea area and just look at its relative depth variation between pixels. Thus, we scale the pixel values of the two images in each sample so that their value range approximately falls into [0,1]∈R. For original (low- or high-resolution) image *I*, its normalized version *I*′ is obtained as follows:

$$\begin{array}{c} I^{\prime }\left(i,j\right)=\frac{I\left(i,j\right)-I_{\text{min}}}{I_{\text{max}}-I_{\text{min}}}, \end{array}$$ (1)

where *i* and j∈Z are horizontal and vertical coordinates of an arbitrary pixel on the image grid, and *I*_min_ and $I_{\text{max}}\in R$ denote the expected minimum and maximum values of *I*, respectively. Here, we use the minimum and maximum values of the low-resolution image to normalize both the low- and high-resolution images and record these values, which enables us to denormalize the estimated high-resolution image back into the original value range by inverting the operation in Eq (1). Note that our normalization is quite different from the normalization commonly performed in superresolution: while general-image superresolution assumes quantized digital images of a known value range (typically [0, 255]) and uniformly processes all samples, we have samples of different value ranges and process each sample independently. Also note that we do not use location information such as latitudes and longitudes in this normalization; for each sample, normalization parameters *I*_max_ and *I*_min_ are determined only from the depth values of the low-resolution image of a certain sea area, but we do not need to know where the area is actually on the globe.

### Network architecture

**Fig 4:**
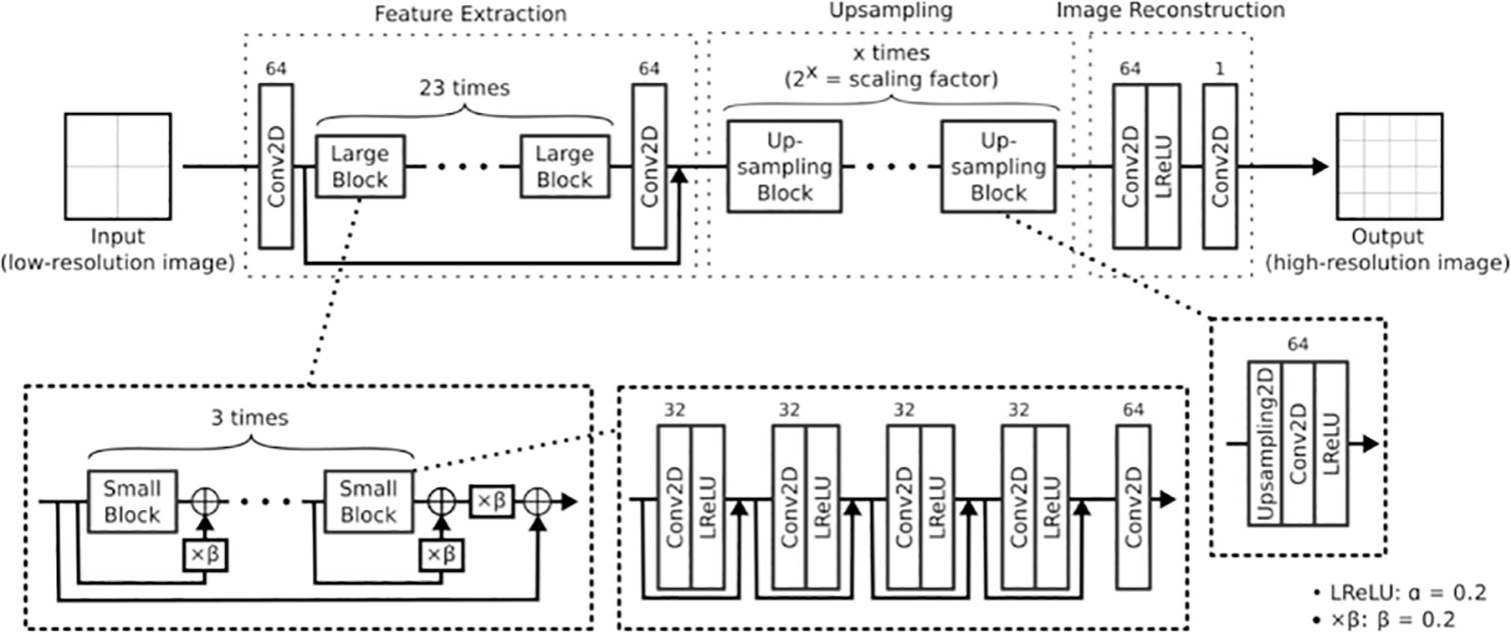
The architecture of the ESRGAN generator [8], i.e., the deep neural network used in the proposed method. Conv2D, LReLU, ×*β* and Upsampling2D denote two-dimensional convolution, leaky rectified linear unit, scaling, and two-dimensional upsampling layers, respectively. The digits on each Conv2D layer indicate its number of filter, while the kernel size is omitted here since it is three for all layers and for both horizontal and vertical axes; also, their strides were one, i.e., convolution is always performed at every pixel.s Branches with simple arrows and arrows pointing at ⊕ denote skip connections with concatenation and addition, respectively. The total number of parameters (weights and biases) is 16,732,609.

As a deep neural network for superresolution, we employ the generator of the state-of-the-art method ESRGAN [8]. Its architecture is depicted in Fig 4. It takes a single low-resolution image as input and yields a high-resolution image as output. This is a very deep network with many layers, and is thus flexible enough to model a complex image transformation, provided that the data are sufficient and learning is successful. All the data in the network, i.e., input and output images, intermediate results (such as image feature maps), and the parameters of the layers are represented as tensors, i.e., multi-dimensional arrays of values.

**Fig 5:**
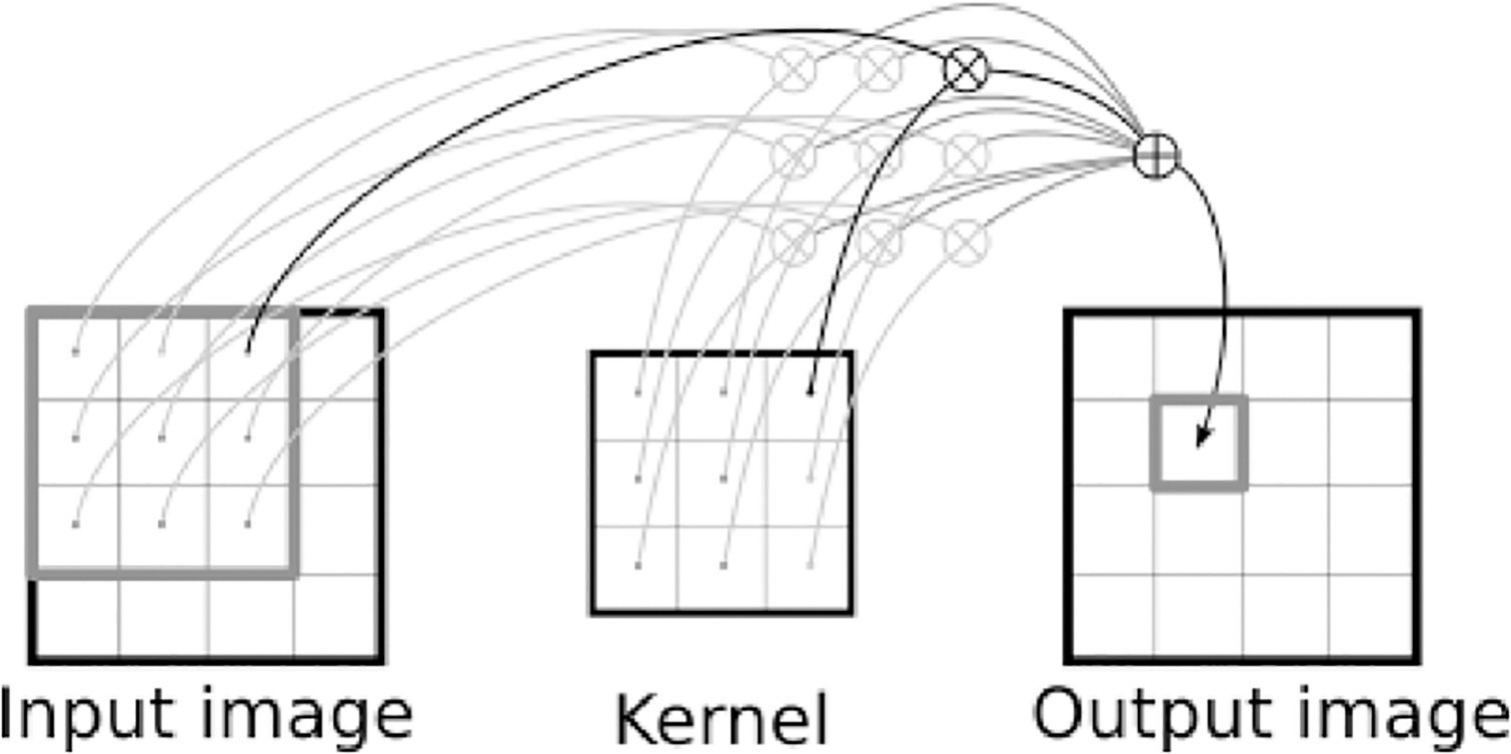
Convolution. ⊗ and ⊕ denote multiplication and addition, respectively. Some operations are grayed out just for visibility. A patch of the input image (whose size is the same as that of the kernel) is multiplied with the kernel pixelwisely, and then summed up into a single pixel of the output image. In reality, the input image may be a single channel of a multi-channel input image; then, multiple intermediate output images are produced with different kernel channels, and then summed into a single output channel of a final output image.

One of the main elements of such an image-processing network is a two-dimensional convolution layer (Conv2D) [13–15], which performs image filtering. Each convolution layer has weight and bias parameters that control what kinds of filters are applied to each input image of this layer to produce an output image. Typically, multiple filters are used, and each filter is associated with a small image with weight values called a kernel, which describes how each patch in the input image is integrated by weighted averaging into a single pixel in the output image, as depicted in Fig 5. In the middle of the network, the input and output images have multiple channels, and so do the kernel; then, each channel of the input image is convolved with the corresponding kernel channel (as in Fig 5), producing multiple intermediate images, which are further summed in the pixelwise manner into a single channel of the final output image. The point is that the same filters (i.e., kernels) are shared among all pixels of the output image, which significantly reduces the number of parameters to be determined and thereby enables efficient learning. Mathematically, let $I\in R^{m\times n\times r}$, $J\in R^{m\times n\times s}$, $W\in R^{p\times q\times r\times s}$, and $B\in R^{m\times n\times s}$ be the input, output, weight, and bias tensors, respectively, where $m,n,p,q,r,s\in Z^{+}$ are the width and height (i.e., the numbers of horizontal and vertical pixel coordinates) of the input and output images, those of the kernel corresponding to the weight, and the numbers of input and output channels, respectively. The convolution operation (multi-channel version of Fig 5) with biasing is defined as follows:

$$\begin{array}{c} J\left(i,j,d\right)=\underset{\text{convolution}}{\underset{︸}{\sum_{c}\sum_{k,l}W\left(k,l,c,d\right)I\left(i+k,j+l,c\right)}}\underset{\text{biasing}}{\underset{︸}{+B(i,j,d)}}, \end{array}$$ (2)

where *i* ∈ {0, …, *m* − 1} and *j* ∈ {0, …, *n* − 1} are the horizontal and vertical coordinates on the images, and *k* ∈ {0, …, *p* − 1} and *l* ∈ {0, …, *q* − 1} are those on the kernel, which correspond to the horizontal and vertical offsets from each pixel location (*i*, *j*), while *c* ∈ {0, …, *r* − 1} and *d* ∈ {0, …, *s* − 1} are the indices for the input and output channels, respectively. Here, *W*(*k*, *l*, *c*, *d*) defines the *d*th one of the *s* kernels, which produces the *d*th output channel via a single multi-channel filtering operation that integrates all input channels; thus, *s* is called the number of filters, whose value for each convolution layer is shown in Fig 4. Meanwhile, for every layer the kernels are of size three, i.e., *p* = *q* = 3, and centered at each pixel (*i*, *j*); hence, their discrete support is defined as *k*, *l* ∈ {−1, 0, +1}. Around image boundaries, where the kernels do not fit inside the input image, the image is appropriately expanded with zero padding, i.e., appending zero-valued pixels.

**Fig 6:**
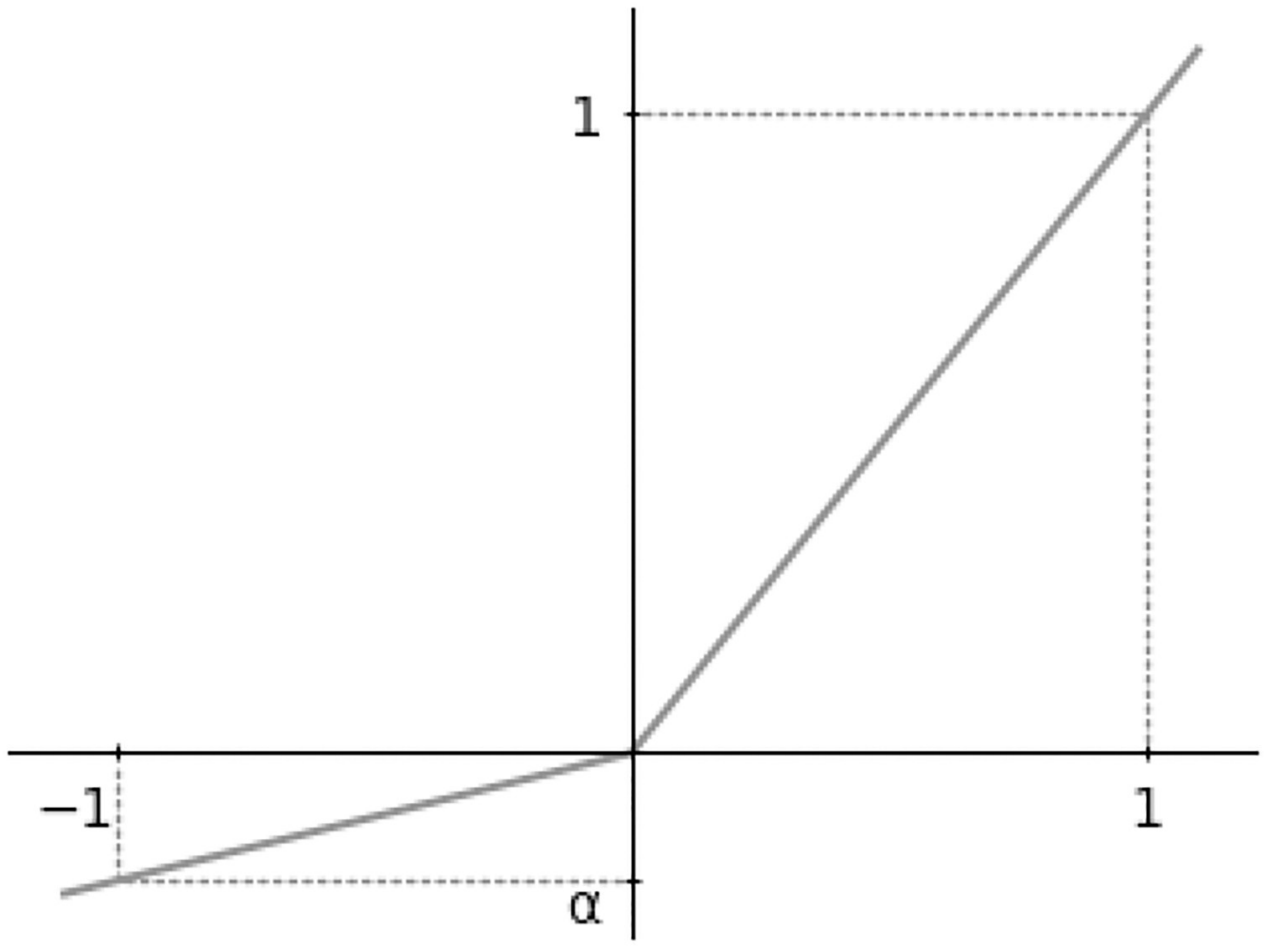
Leaky Rectified Linear Unit (LReLU). The parameter *α* controls the amount of negative-value leaking.

Another important element of neural networks is an activation layer, which performs an elementwise nonlinear transformation. The role of this layer is to modify the values after the linear transformation by the convolution layers and thereby enable the network to represent an arbitrary nonlinear transformation. Specifically, the ESRGAN network uses the leaky rectified linear unit (LReLU) function [16] as the activation layer, which is depicted in Fig 6 and defined as follows:

$$\begin{array}{c} f\left(x\right)=\{\begin{array}{cc} x & \text{if}\;x>0\\ \alpha x & \text{otherwise} \end{array}, \end{array}$$ (3)

where the parameter *α* controls the amount of negative-value leaking. This operation is performed in the elementwise manner, i.e., it is applied to each scalar value of the input tensor to this layer (typically the output tensor of a convolution layer). This function lets positive values pass through, but attenuates negative values to zero while allowing some information to leak, which facilitates smooth learning. In the employed network, the hyperparameter *α* of the LReLU activation layers is fixed to 0.2.

The first part of the ESRGAN network aims to extract image features, which should implicitly represent geometric structures like edges. Four pairs of convolution and activation layers are chained and followed by another convolution layer to form a small block, which is further repeated three times to construct a large block. In total, the feature-extraction part consists of 23 large blocks, each of which is composed of three small blocks. Moreover, the network has skip connections, each of which feeds the input of a layer to the output of another layer so that only the residual (difference) from the input and the expected output needs to be produced in the skipped part, reducing the amount of information to be learned and thereby facilitating the use of many layers [17]. While most of the skip connections simply concatenate input tensors along the channel axis, those between the small blocks perform addition (⊕) equipped with residual scaling (×*β*), a technique that weakens the magnitude of the residual to stabilize learning [18]; here, the residual is multiplied by *β* = 0.2. In combination, these operations transform the input low-resolution image into a set of feature maps, each of whose pixel value implicitly indicates the existence of a local image structure such as an edge.

**Fig 7:**
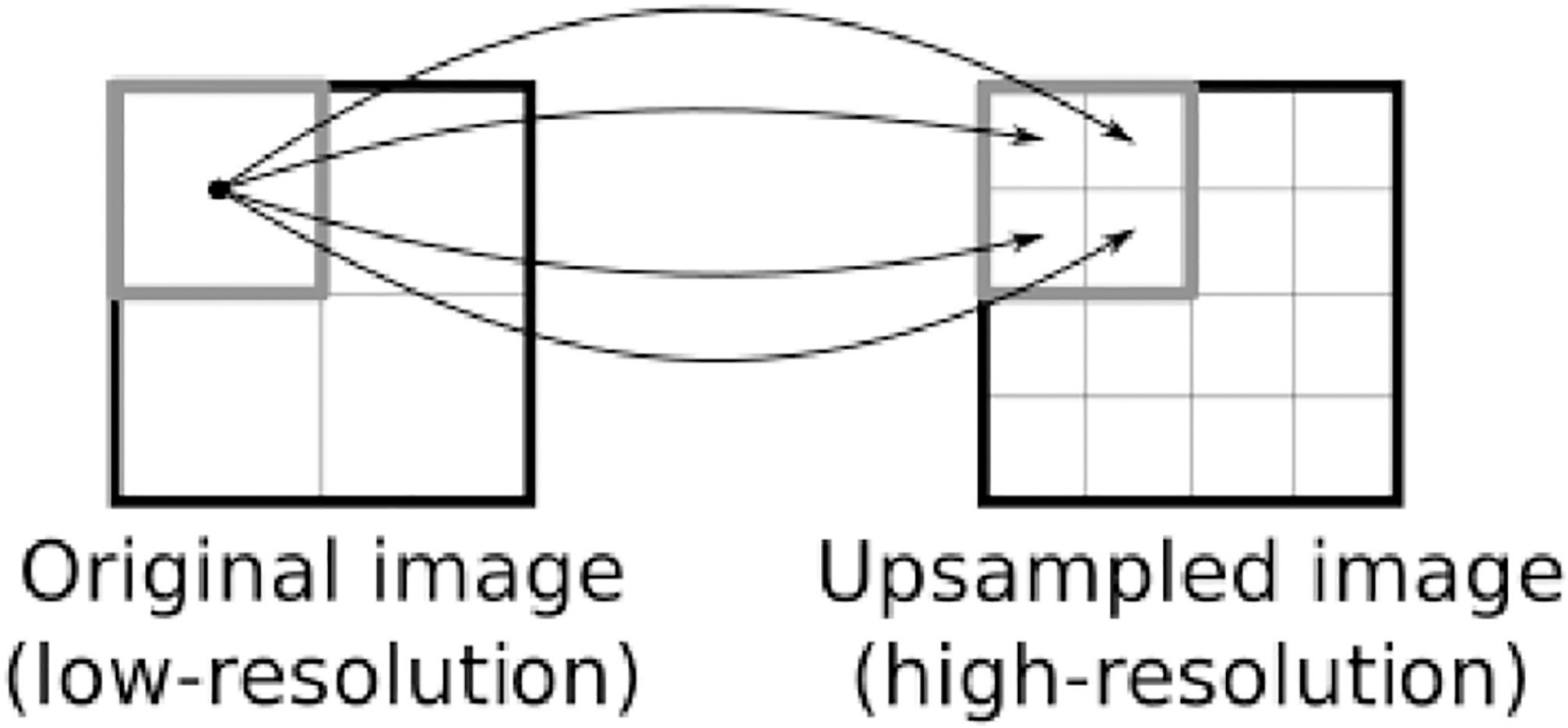
Two-dimensional upsampling by nearest-neighbor interpolation. The arrows indicate copying of pixel values. While only the top-left pixel of the original image is considered here, every pixel is processed in the same manner in the actual operation.

Then, the feature maps are enlarged by upsampling, at which point the resolution is enhanced from low to high. This is done by first doubling the size of each map by spatial nearest-neighbor interpolation with a two-dimensional upsampling layer (Upsampling2D), whose operation is depicted in Fig 7. For example, if we perform a superresolution operation of scaling factor two, one pixel of the low-resolution input becomes four pixels (arranged on a two-by-two grid) in the high-resolution output. In the nearest-neighbor case, the value of the input pixel is simply copied to the output pixels. Then, upsampled pixel values are mixed by a pair of convolution and activation layers to yield high-frequency information. With larger scaling factors such as four and eight (required to be powers of two for easy computation), we just repeat this process by adding upsampling blocks. Owing to the use of the learned image features, this upsampling process in the image-feature domain can recover image structures better than can naive upsampling in the raw image domain.

Finally, a single high-resolution image is reconstructed from the upsampled feature maps via additional convolution and activation layers. At this point, we supplement the original ESRGAN architecture with the common superresolution technique called residual learning [19], which lets the network produce only the difference between an upsampled version of the low-resolution image and the expected high-resolution image, thereby reducing the amount of information to be learned. We realize this simply by inserting an additive skip connection between the input upsampled by bicubic interpolation and output of the network.

### Training

When training the network, we adjust its parameters so that it correctly estimates the high-resolution image corresponding to each low-resolution image in the dataset. More specifically, we minimize a loss function that is defined as the total error between all pairs of the high-resolution image estimated from the low-resolution images and the corresponding true high-resolution images, i.e.,

$$\begin{array}{c} L\left(G\right)=\sum_{\left(I_{\text{LR}},I_{\text{HR}}\right)}∥I_{\text{HR}}-G\left(I_{\text{LR}}\right)∥, \end{array}$$ (4)

where *G* is the network (superresolution estimator) to be optimized with respect to, (*I*_LR_, *I*_HR_) is the pair of low- and high-resolution images as a sample in the dataset, respectively, and ‖⋅‖ denotes the L1 norm. Note that we calculate the loss between two high-resolution images *I*_HR_ and *G*(*I*_LR_) (not low-resolution *I*_LR_), whose number of pixels is the same. The use of the L1 norm (absolute error) instead of the L2 (squared error) [8] is for robust estimation, where outliers like large noise in the low-resolution observation do not affect the high-resolution estimation.

The optimization of the loss function can be done iteratively by the technique called stochastic gradient descent, which uses the gradient of the loss function *L* with respect to the parameters of *G* (in our case, the weights and biases of the convolutional layers) to determine the directions in which the parameter values should be moved at each iteration. Although the network is made of many layers and is thus a complicated function, the gradient with respect to the parameters of an arbitrary layer is available owing to the common deep-learning technique called backpropagation, which propagates the error values required for gradient computation from the output to the input of the network. Specifically, we employ the Adam optimizer [20], which can automatically adjust the learning rate, i.e., the amount of parameter modification at each iteration, to ensure the convergence of the iterative optimization; here, we use Adam with default parameters *β*_1_ = 0.9 and *β*_2_ = 0.999, and initial learning rate 10^−4^. Due to the limited capacity of computer memory, the actual optimization is performed for each batch, i.e., a subset of the dataset consisting of several samples, rather than the whole dataset, and is iterated over all batches. A single pass over the entire dataset (processing all the samples) is called an epoch, and we perform iterations of 100 epochs, wherein convergence is empirically observed.

### Implementation

We implement the proposed method using the Python programming language and the Keras machine-learning library [21]. Keras provides neural network layers, e.g., convolution and activation layers, as functions, and automatically computes the gradients required for optimization, thereby allowing users to easily implement a deep-learning method. Moreover, it automatically translates all operations so that the program runs on a graphics processing unit (GPU), which enables fast computation during the training of a deep neural network with a large amount of data.

## Results and discussion

To evaluate the effectiveness of the proposed method, we conducted an experiment on bathymetric image superresolution.

For the evaluation data used in this experiment, we used publicly-available GEBCO gridded bathymetric data [1]. Specifically, we used GEBCO 2014 and 2019 data, whose resolutions are 30 arc-seconds (approximately 900 meters) and 15 arc-seconds (approximately 450 meters), as low-resolution input and high-resolution output images, respectively. From these two sets of data, we first cropped corresponding sea areas with no overlaps around Japan, each of which is a square with sides of length 3,840 arc-seconds in latitude or longitude (approximately 115 kilometers); here, each area was cropped only if it resided within the range between (N 20;25;31, E 122;55;57) and (N 45;33;26, E 153;59;12), with its corners’ latitudes and longitudes being powers of 3,840 arc-seconds, and contained no lands (i.e., positive elevation values) in both of the low- and high-resolution sets. Consequently, we obtained 434 samples, each of which consists of the low- and high-resolution images covering the same area, and the sizes of these low- and high-resolution images were 128 × 128 and 256 × 256 pixels, respectively. Note that we used GEBCO data based on real measurement for both high-resolution outputs and low-resolution inputs; this is different from the standard experimental scheme in learning-based image superresolution [8], where artificially downsampled versions of high-resolution images are used as low-resolution images, which might have different statistical properties from real observations. Since the learning-based method could remember learned samples and perform unrealistically accurate prediction for the same samples, in the interest of a fair evaluation, we split these samples for training and testing, in which 80% and 20% of the samples were used, respectively. We then applied the data-preprocessing techniques to these samples as described in the Data preprocessing section. It should be noted that data augmentation was performed only for the training samples involved in learning, while normalization was also applied to the testing samples in order to match the value ranges between training and testing. In total, the number of training samples was 2,776 (347 × 8) after data augmentation, while that of the testing samples was 87.

In this experiment, we compared the proposed deep-learning-based superresolution method with conventional bicubic interpolation as a baseline method. Note that this interpolation was performed on the low-resolution image of each sample, i.e., the same input as in the case of the proposed method. For the proposed method, we first let the network learn the training samples, as described in the Training section. Then, we estimated the high-resolution image from the corresponding low-resolution image of each testing sample by the baseline and proposed methods.

**Fig 8:**
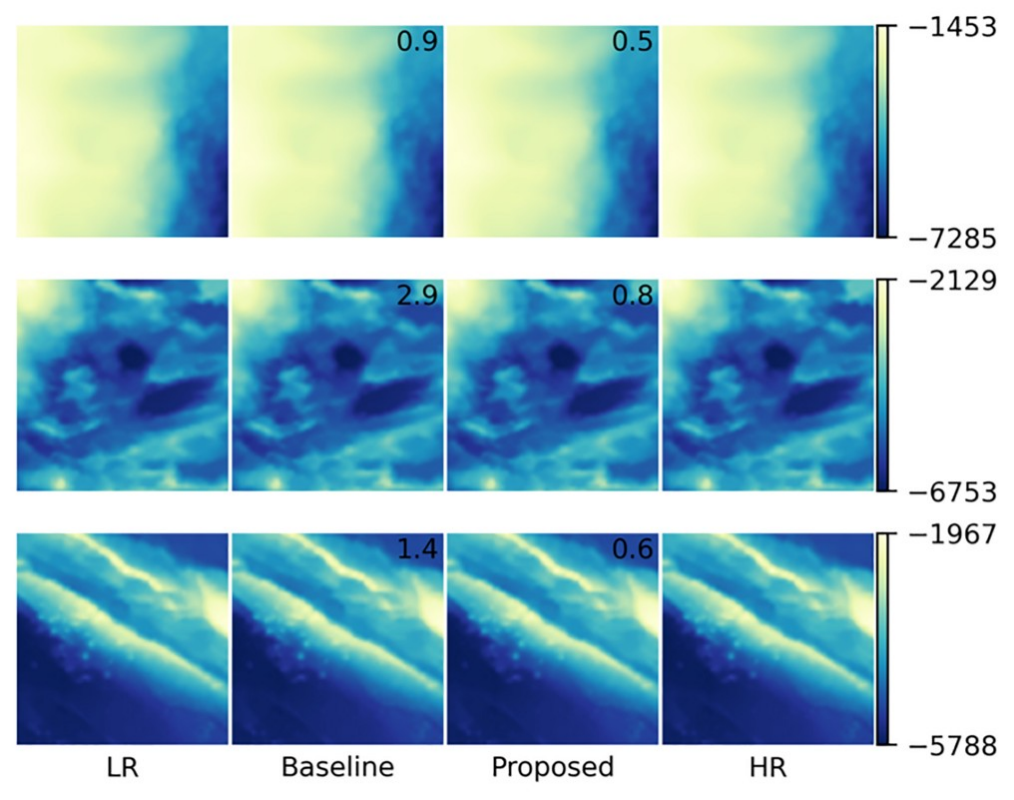
Visualized images of three testing samples as the results of superresolution. Each row corresponds to a single testing sample, and the colorbar on the left of the row shows its value range. The columns labeled LR, Baseline, Proposed, and HR correspond to input low-resolution, output high-resolution estimated by the baseline and proposed methods, and true high-resolution images, respectively. The numbers in the estimated images (Baseline and Proposed) represent the root mean squared error (RMSE) values against the corresponding true high-resolution images (HR). Note that these values are not the average over all samples in our dataset, but the square roots of the mean squared errors over all pixels in each image (column) for each sample (row). The colorbar for each sample indicates its depth value range in meters. Note that the value ranges and the RMSE values were computed after denormalizing the images.

**Fig 9:**
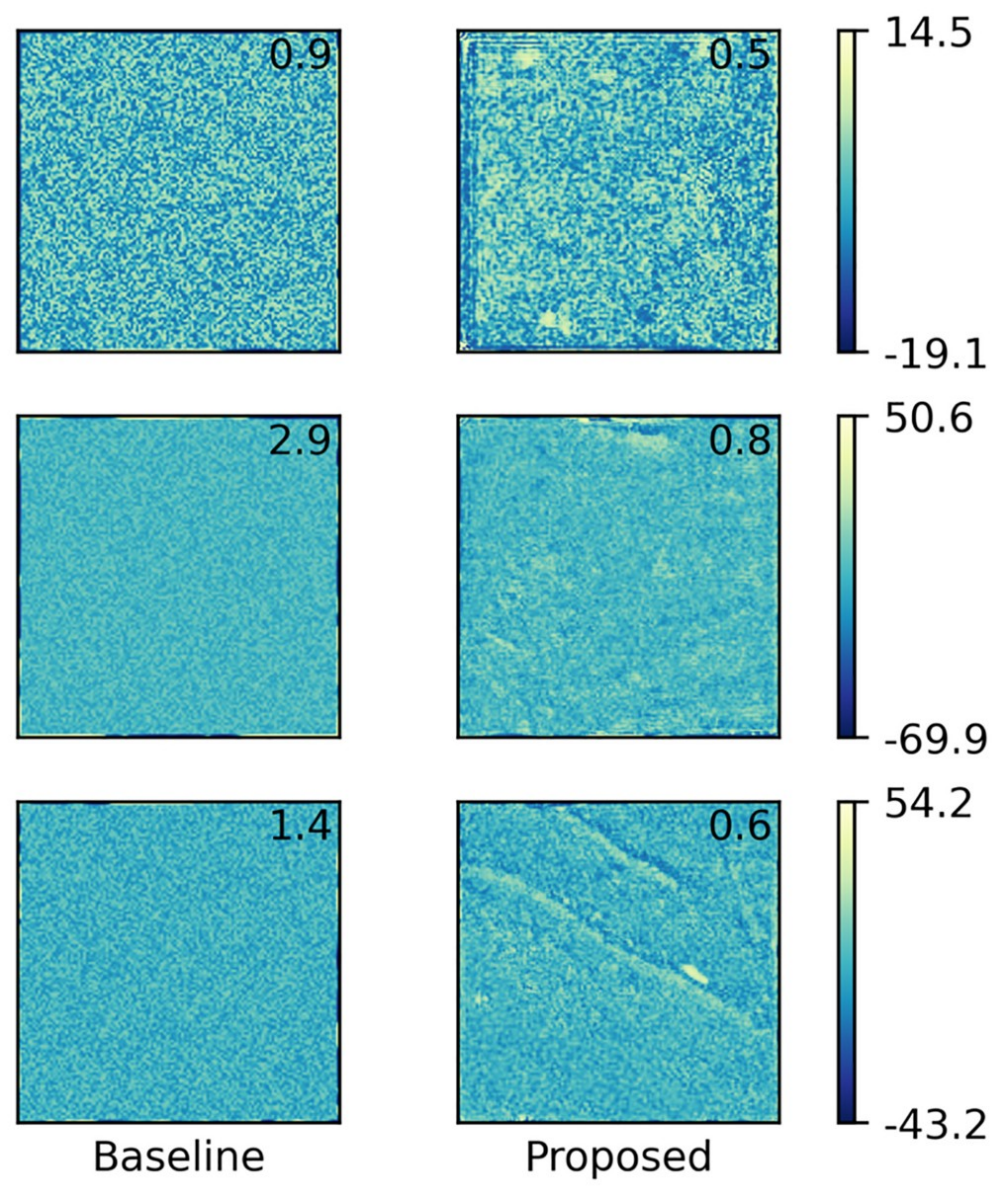
Differences of estimated images by the baseline and proposed methods from the corresponding true high-resolution images in Fig 8. For visibility, symmetrical logarithmic scaling is employed in coloring. The number in each image represents the corresponding RMSE values (i.e., the root mean square of its pixel values).

**Fig 10:**
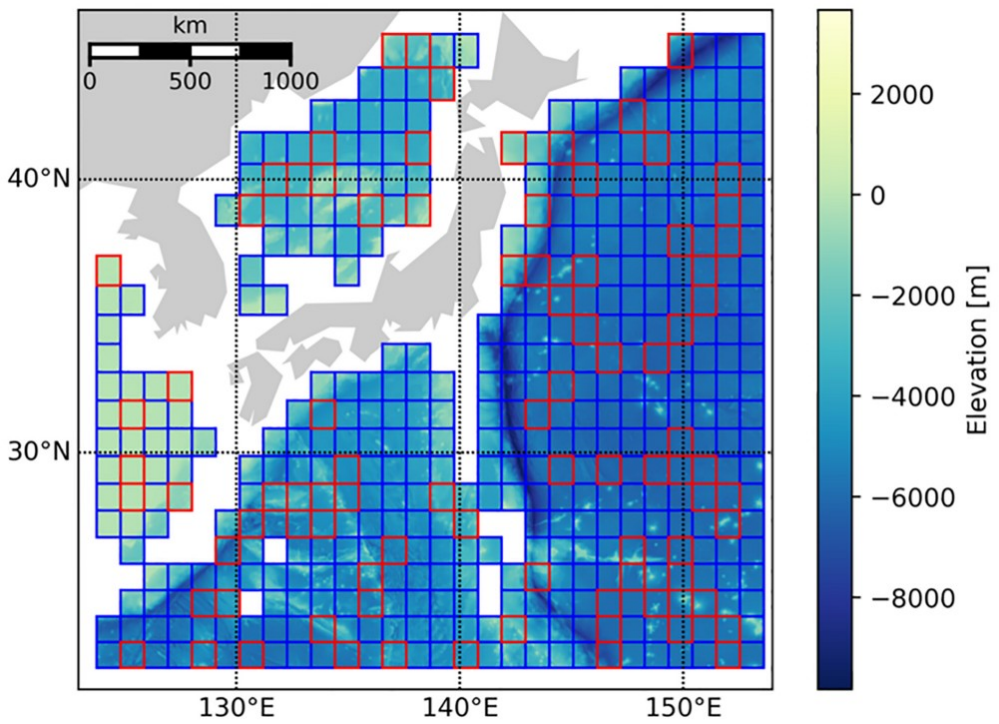
High-resolution bathymetric map predicted by the proposed method. Blue and red boxes contain estimated high-resolution images in the training samples (347 areas) the testing samples (87 areas), respectively.

To qualitatively assess the quality of the estimated high-resolution images in comparison with the corresponding true high-resolution image as ground truth, we visualized the resulting images for testing samples corresponding to three areas by coloring their depth values, as shown in Fig 8; here, to enable quantitative comparison, the root mean squared error (RMSE) values against the true high-resolution image are also shown. From the colorbars, we can see that the value ranges varied a lot between samples, depending on their absolute depths, i.e., whether their sea areas are deep or shallow. Moreover, the proposed method achieved smaller RMSE values than the baseline method regardless of sample, which indicates its higher performance quantitatively. This can be visually confirmed by looking at the difference images of the results with respect to the ground truth summarized in Fig 9; here, while both methods produced erroneous pixels distributed almost uniformly over each area, the baseline method produced large errors near area boundaries, which are inherent in interpolation and can be problematic in practice when we have real measurements only at limited locations. On the other hand, the proposed method yielded more structured errors, which can be attributed to the property of CNNs that it may sometimes produce fake structures as artifacts [22]; nevertheless, the produce method produces smaller errors than the baseline in total, as indicated by the RMSE values. Actually, the proposed method outperformed the baseline for all the 87 testing samples in terms of RMSE. This also demonstrates the robustness of the proposed method against the variation in value ranges, owing to the normalization in our data preprocessing. To summarize the results, Fig 10 shows the high-resolution bathymetric map predicted by the proposed method for the whole sea areas considered in this experiment. Overall, the proposed method successfully produced smoothly-continued superresolution results between the areas belonging to training (blue boxes) and testing (red boxes) data without actually referring to the training samples in testing, which indicates that the network properly learned the geometry of the bathymetric image data.

To measure the total performance of the two methods, we used the peak signal-to-noise ratio (PSNR), a standard image quality metric that summarizes the pixelwise error between true and estimated images. In addition, we used structural similarity (SSIM) [23], which is another popular image quality metric that is intended to better reflects differences in image structures like edges. Here, we computed these metrics from normalized images, whose values mostly fall into [0, 1] and are clipped when out of the range. Note that we do not use denormalized metrics such as RMSE here, since they vary a lot between samples (as seen in Fig 8), and thus, do not produce meaningful values when averaged over samples. The average PSNR and SSIM values and their standard deviation over all testing samples is presented in Table 1; here, higher PSNR and SSIM values correspond to better image qualities. Overall, the proposed method was able to estimate high-resolution bathymetry much more accurately than the baseline method in terms of both PSNR and SSIM. Furthermore, the performance of the proposed method is analyzed in more details in Table 2 for sea areas in different depth ranges. Here, we categorized the testing samples into three depth ranges, i.e., shallow, medium, and deep, by their average depth values in the true high-resolution images. These ranges were defined using 3-quantiles of the average values over all testing samples to make the numbers of samples even between ranges. It shows that the proposed method yielded the best performance in the medium range.

**Table 1:** Average PSNR and SSIM ± standard deviation over all testing samples.

Metric	Baseline	Proposed
PSNR [dB]	40.7 ± 12.9	41.6 ± 14.4
SSIM	0.951 ± 0.069	0.954 ± 0.064

**Table 2:** Average PSNR of the proposed method over the testing samples in different depth ranges.

Range	Depth [m]	PSNR [dB]
Shallow	*d* < 4, 379	39.9
Medium	4, 379 ≤ *d* ≤ 5, 588	47.8
Deep	*d* > 5, 588	37.2

The depth *d* of each sample is defined as the average depth of the true high-resolution image. The range boundary depths 4,379 and 5,588 correspond to the 3-quantiles of the average depths over all testing samples.

According to the previous study on the learning-based bathymetric image superresolution, the best-performing traditional (i.e., non-deep) learning-based method was random forest [11]. While the authors of this previous study used a different dataset and a scaling factor of six, we can compare their results with ours by focusing on the PSNR improvement over the results of bicubic interpolation. In Table 1, the proposed method scored a PSNR improvement of approximately one dB over the bicubic interpolation, which is similar to the reported performance of their random-forest method [12]. However, the performance of bicubic interpolation on their dataset was approximately 45 dB, which is higher than our case (about +4 dB) and thus their problem difficulty could be much easier than our case. Therefore, we could expect higher performance of the proposed method than the previous one with the same experimental setting. In addition, using our dataset, we tested a random-forest-based general-image superresolution method [24], on which the previous bathymetric-image method [12] was based and whose implementation was publicly available. Since this implementation assumed general images with a fixed value range, we fed the [0, 1]-normalized low-resolution images in our dataset as inputs. The resulting average PSNR of this method over all testing samples was 37.5 dB, which was much lower than the proposed method (about -4 dB) and even worse than the bicubic baseline method according to Table 1. This might be because the traditional approach could not fully deal with the large amount of data with complex geometry like bathymetric data, although the non-deep-learning method has fewer parameters and thus theoretically needs less training data for learning than the proposed method. We note that the learning ability of such non-deep-learning-based methods may be compensated for by introducing handcrafted prior knowledge or domain-specific tuning, e.g., the variance-modification trick used in the previous study [12], although the reported PSNR improvement by this trick was only about 0.3 dB, which illustrates the limitation of such a non-data-driven heuristic approach. Therefore, the proposed method was more effective than this random-forest method, demonstrating the superiority of deep learning over traditional learning. Another problem with the random-forest method is its high computational demand, i.e., its MATLAB CPU implementation required all training data to be expanded onto computer memory, thus being inapplicable to huge amounts of data or environments with limited computational resource. In contrast, the proposed method is trained via batchwise optimization, where only a small number of samples (eight in our experiment) in the training dataset need to be loaded onto memory at each iteration. Although the deep-learning approach takes more training time due to its iterative nature (approximately 14 minutes per epoch on average in our experiment using a NVIDIA Tesla V100 PCIe GPU), this can be regarded as the price of its effectiveness and memory efficiency (note that we can use a larger batch size to accelerate the training if memory restriction can be relaxed). Moreover, as discussed in the Implementation section, such a neural network can be efficiently implemented for GPUs using openly-available, widely-used, and well-documented libraries. Hence, the proposed method is easier to deploy both computationally and technically such a traditional method, which is another advantage of the deep learning approach.

Additionally, to validate the effectiveness of our data preprocessing for bathymetric data, we show the PSNR performance of the proposed method with and without the two preprocessing techniques, i.e., augmentation and normalization in Table 3. This shows that combining the two techniques led to the best performance. Hence, we can tell that our assumptions made for the two preprocessing techniques (i.e., flip-and-rotation invariance and depth independence) are valid for actual bathymetric data, and both of them are crucial given the limited amount of bathymetric data.

**Table 3:** Average PSNR [dB] by the proposed method over all testing samples for scaling factor two with and without data-preprocessing techniques.

Normalization	No	Yes
Augmentation		
No	41.0	40.8
Yes	41.4	41.6

The full version of the proposed method is with both normalization and augmentation (the bottom-right value, which is from the same trial as the top-right value in Table 1).

Finally, we analyzed the performance of the proposed method for different image sizes. Note that varying the size of images in our experimental setting also changes the number of samples, as shown in Table 4, because a larger image size reduces the sea areas that can be cropped without overlapping lands, thereby descreasing the amount of data (i.e., the number of pixels) available for training. Despite this, the best PSNR score was observed at the largest image size (128 × 128 pixels for low-resolution input). This result indicates that larger input images are desirable for the proposed convolutional network to capture maximum geometric information, even if the amount of training data are compromised.

**Table 4:** The average PSNR of the proposed method over the testing samples for different image sizes.

Image size [pixels]		Number of samples		PSNR [dB]
Low-resolution	High-resolution	Training	Testing	
128 × 128	256 × 256	347	87	41.6
64 × 64	128 × 128	1613	403	40.8
32 × 32	64 × 64	7005	1751	36.5

The numbers of training samples were calculated before data augmentation.

## Conclusion

In this work, we proposed to use deep-learning-based image superresolution to enhance the resolution of bathymetric data. Specifically, we made use of a deep neural network and preprocessing techniques such as data augmentation and normalization to effectively resolve features specific to bathymetric images. Through an experiment, we confirmed that the proposed method can effectively generate high-resolution bathymetric images of higher quality than those produced via conventional interpolation.

Although we only used samples from sea areas around Japan, the performance of the proposed method should improve as more data become available for learning. Performance analysis in other sea areas (e.g., the east coast of the United States) and with specific sensors (unlike the NOAA and GEBCO data, which are collections of measurements from various sources) are also future subjects, while the accuracy of the network can be further improved by fine-tuning its parameters using new training samples in such cases. Satellite data may be also useful as additional training data to widen the range of the sea areas that the network can cope with, especially in shallower seas where higher resolutions are desired to capture steep depth slopes. Other possible future directions include transfer learning from topographic (land) data into bathymetric (ocean) data, exploiting their similarity to compensate for the scarcity of bathymetric data. The direct use of raw depth-measurement data is also of interest, since the original pointwise data may contain more high-frequency information than already-gridded data; such a problem of reconstructing dense grid data from sparse point data may also be addressed by importing recent knowledge from general-image processing [25] into bathymetry. Finally, it is possible to quantify the uncertainty in superresolution estimation using statistical frameworks, which enables us to find sea areas where superresolution estimation may be relatively unreliable and thus real observation would be particularly valuable. This would allow us to realize efficient seafloor mapping via adaptive measurement, which limits dense depth measurement to the sea areas where superresolution based on the existing data has difficulty recovering details from coarse data. Such a hybrid approach to bathymetry, i.e., the combination of superresolution estimation and minimal observation, will significantly accelerate the creation of high-resolution bathymetric charts of the world’s oceans. Note that our methodology is not computationally restricted even in processing a large amount of global data, since such data can be split into small batches to be fed into the network. Moreover, additional data can be learned incrementally upon new measurement. This learning process does not need to be performed in real time, and prediction after learning is fast because it only requires a single forward pass of given low-resolution measurements through the network. At the same time, it will also add to the amount of available bathymetric data on the seafloor patterns that the currently-available data do not describe well, and further enhance the effectiveness of the proposed learning-based method.
